# Galangin Resolves Cardiometabolic Disorders through Modulation of AdipoR1, COX-2, and NF-κB Expression in Rats Fed a High-Fat Diet

**DOI:** 10.3390/antiox10050769

**Published:** 2021-05-12

**Authors:** Patoomporn Prasatthong, Sariya Meephat, Siwayu Rattanakanokchai, Juthamas Khamseekaew, Sarawoot Bunbupha, Parichat Prachaney, Putcharawipa Maneesai, Poungrat Pakdeechote

**Affiliations:** 1 Department of Physiology, Faculty of Medicine, Khon Kaen University, Khon Kaen 40002, Thailand; pa_pra@kkumail.com (P.P.); sariya_m@kkumail.com (S.M.); juthakh@kku.ac.th (J.K.); putcma@kku.ac.th (P.M.); 2 Faculty of Veterinary Medicine, Khon Kaen University, Khon Kaen 40002, Thailand; siwara@kku.ac.th; 3 Faculty of Medicine, Mahasarakham University, Maha Sarakham 44000, Thailand; sarawoot.b@msu.ac.th; 4 Department of Anatomy, Faculty of Medicine, Khon Kaen University, Khon Kaen 40002, Thailand; parpra@kku.ac.th; 5 Research Institute for Human High Performance and Health Promotion, Khon Kaen University, Khon Kaen 40002, Thailand

**Keywords:** galangin, cardiac function, metabolic syndrome, oxidative stress, inflammation, adiponectin

## Abstract

Galangin is a natural flavonoid. In this study, we evaluated whether galangin could alleviate signs of metabolic syndrome (MS) and cardiac abnormalities in rats receiving a high-fat (HF) diet. Male Sprague–Dawley rats were given an HF diet plus 15% fructose for four months, and they were fed with galangin (25 or 50 mg/kg), metformin (100 mg/kg), or a vehicle for the last four weeks. The MS rats exhibited signs of MS, hypertrophy of adipocytes, impaired liver function, and cardiac dysfunction and remodeling. These abnormalities were alleviated by galangin (*p* < 0.05). Interleukin-6 and tumor necrosis factor-α concentrations and expression were high in the plasma and cardiac tissue in the MS rats, and these markers were suppressed by galangin (*p* < 0.05). These treatments also alleviated the low levels of adiponectin and oxidative stress induced by an HF diet in rats. The downregulation of adiponectin receptor 1 (AdipoR1) and cyclooxygenase-2 (COX-2) and the upregulation of nuclear factor kappa B (NF-κB) expression were recovered in the galangin-treated groups. Metformin produced similar effects to galangin. In conclusion, galangin reduced cardiometabolic disorders in MS rats. These effects might be linked to the suppression of inflammation and oxidative stress and the restoration of AdipoR1, COX-2, and NF-κB expression.

## 1. Introduction

Metabolic syndrome (MS) is comprised of a set of cardiometabolic risk factors such as dyslipidemia, insulin resistance, central obesity, and hypertension. The presence of three or more specific factors is indicative of MS, which contributes to the development of cardiometabolic disease and type 2 diabetes [1]. It is well documented that excessive caloric intake is the major cause of obesity and metabolic syndrome in humans [2]. Epidemiological studies provide substantial evidence linking dietary consumption patterns to the development of obesity and MS [3]. Obesity has been proposed to be the cause of adverse effects on the metabolic system, dyslipidemia, and hyperglycemia. In a rat model of MS, HF diet-induced metabolic disturbances have been associated with signs of MS and cardiovascular alterations in humans [4]. Several studies have demonstrated that rats that received an HF diet had hyperglycemia, an impaired oral glucose tolerance test (OGTT), hyperinsulinemia, dyslipidemia, visceral fat pad accumulation, and hypertension [5,6]. Additionally, fructose supplementation in rats can induce signs of MS, as has been documented [7,8]. Numerous studies indicated that diet-induced MS in animals also shows the characteristics of cardiac alterations including the impairment of cardiac function and morphology. For example, Ouwens and coworkers demonstrated changes in cardiac phenotype in rats receiving an HF diet, supported by cardiac hypertrophy and dysfunction associated with myocardial triacylglycerol accumulation [9]. A previous study demonstrated that rats fed an HF diet plus fructose in drinking water had impaired glucose tolerance, dyslipidemia, visceral fat accumulation, and hypertension associated with left ventricular (LV) hypertrophy and dysfunction [10].

Chronic-low-grade inflammation has been found in MS associated with hypertrophy of adipocytes or visceral fat accumulation, alongside several proinflammatory cytokines including interleukin-6 (IL-6), tumor necrosis factor-α (TNF-α), interleukin-8 (IL-8), leptin, etc. [1,11]. This local inflammation in adipocytes can produce systemic inflammation and a progression of cardiovascular and metabolic disease [12,13]. On the contrary, a reduction of adiponectin levels has been observed in obesity, and this adipokine exhibits inhibitory inflammatory processes [14]. Adiponectin has been recently described to be an anti-inflammatory and cardioprotective cytokine as low levels of adiponectin have been revealed in patients with severe coronary artery disease (CAD) and left ventricular hypertrophy with diastolic dysfunction [15,16,17]. The cardioprotective effects of adiponectin are associated with suppressing reactive oxygen species-induced cardiac remodeling in rats [18]. Additionally, the adiponectin/adiponectin receptor 1 (AdipoR1) signaling pathway has a crucial role in the regulation of mitochondrial function, oxidative stress, and lipid and glucose metabolism in the muscles of mice [19]. AdipoR1 is also mainly expressed in heart and mediates the preventive effect of adiponectin on myocardial injury induced by ischemia–reperfusion (I/R) through enhancing cyclooxygenase-2 (COX-2) [20]. It is recognized that COX-2 is detrimental and plays a role in inflammatory processes. However, anti-inflammatory and cardioprotective effects of COX-2 have been described [21,22]. There is evidence showing that nuclear factor kappa B (NF-κB), a transcriptional factor, is suppressed by the adiponectin/AdipoR1 signaling pathway [23]. NF-κB has a deleterious effect on heart because the blockade of NF-κB can alleviate cardiac failure and remodeling in knockout mice with myocardial infarction [24]. Furthermore, oxidative stress has been characterized as contributing to the development of cardiometabolic disease in rats with diet-induced MS [25,26,27]. There is growing evidence that MS rats have high levels of local and systemic oxidative stress biomarkers and malondialdehyde (MDA) and low activities of endogenous antioxidant enzymes, superoxide dismutase (SOD), and catalase (CAT) [28,29]. Adipose tissue has been suggested to be a main source of free radical in obese mice [30]. In contrast, adiponectin signaling has been proposed to reduce reactive oxygen species [23].

It is widely suggested that the initial management of MS involves lifestyle modifications, including changes in diet and exercise habits. Metformin is a biguanide family member and is recommended for type 2 diabetes treatment. The hypoglycemic effects of metformin are relevant to improving the insulin sensitivity of liver and peripheral tissue and reducing hepatic glucogenesis [31]. It also improves the lipid profiles in MS rat induced by diet [32]. Metformin has been noted to reduce inflammatory markers in diabetic rats fed high levels of fructose [33]. Moreover, the beneficial effects of metformin have been documented in patients with CAD, as it reduces systolic blood pressure (SBP), left ventricular (LV) hypertrophy, LV mass indexed, body weight (BW), and oxidative stress [34]. In this study, metformin was a positive control substance to mitigate cardiometabolic syndrome in HF diet-induced MS rats.

Currently, several studies have revealed the advantageous effects of flavonoids in terms of reducing the severity of disease together with drugs. Galangin (3,5,7-trihydroxyflavone or 3,5,7-trihydroxy-2-phenyl-4H-chromen-4-one) is a natural flavonoid that is mostly found in honey, Alpinia officinarum Hance (Zingiberaceae), and the rhizome of Alpinia galanga. Several biological effects of galangin have been demonstrated, such as antimicrobial [35], antitumor [36], anti-apoptotic [37], antifibrotic [38], and anti-inflammatory activities [39]. In an animal model of type I diabetes mellitus, streptozotocin (STZ)-induced diabetic rats, galangin alleviated oxidative stress by increasing the activities of endogenous antioxidant enzymes such as SOD, CAT, glutathione peroxidase, and glutathione-S-transferase [40]. It also ameliorated hyperglycemia, hyperinsulinemia, and dyslipidemia in rats treated with STZ [41]. Recently, galangin has exhibited hepatoprotective effects via the activation of nuclear factor erythroid 2-related factor 2 and the heme oxygenase 1 signaling pathway in cyclophosphamide-administered rats [42]. However, little information regarding the effect of galangin on cardiometabolic disorders in a model of rats with HF diet-induced MS has been shown. In this study, we evaluated whether galangin could alleviate the signs of MS, cardiac alterations, oxidative damage, and inflammation induced by an HF diet in rats.

## 2. Materials and Methods

### 2.1. Animals

Male 6-week-old Sprague–Dawley rats weighing 200–220 g were purchased from Nomura Siam International Co., Ltd., Bangkok, Thailand. All rats were housed in standard cages in a temperature-controlled room (23 ± 2 °C) with a relative humidity of 30–60% and a light/dark cycle of 12 h. All procedures were performed in accordance with the rules of the ethical guidelines for the Care and Use of Laboratory Animals, which was approved by the Animal Ethics Committee of Khon Kaen University (IACUC-KKU-74/62), based on the ethical animal experimentation guidelines of the National Research Council of Thailand. All rats had free access to food and water. A standard chow diet and an HF diet were used to feed control rats and MS rats, respectively. The standard chow diet was composed of 57.81% carbohydrates, 22.9% protein, and 5.72% fat, while the HF diet was composed of 46.3% carbohydrates, 13.25% protein, and 24.29% fat. The composition of the standard chow and HF diet were analyzed by Central Lab Thai (Central Laboratory (Thailand) Company Limited, Khon Kaen, Thailand). The control rats were given tap water, while the MS rats were supplemented with fructose (15%) in drinking water during the night to facilitate signs of MS.

### 2.2. Research Designs

After acclimatization, control rats were given a standard chow diet and tap water for 16 weeks (*n* = 8). MS rats were given an HF diet and fructose (15%) in drinking water for 16 weeks. After 12 weeks of the experiment, MS rats were subdivided into four groups (*n* = 8/group): MS rats that were given a vehicle, MS rats that received galangin (25 mg/kg), MS rats that received galangin (50 mg/kg), and MS rats that received metformin (100 mg/kg). Galangin (purity ≥98%) was purchased from Aktin Chemicals, Inc. (Mianyang City, Sichuan, China). Metformin was purchased from Siam Pharmaceutical Company Ltd. (Bangkok, Thailand). All treatments were performed by oral administration using a gavage tube daily for the final four weeks of the experiment period. At the 12th week, all rats were fasted overnight to collect blood samples via the lateral rat tail vein for lipid profile and blood glucose measurement to confirm the characteristics of MS. The concentration of galangin was influenced by the pilot study.

### 2.3. Measurement of Systolic Blood Pressure

Conscious rats were evaluated monthly in terms of their SBP changes during the three months of the experimental period and weekly during the final four weeks of treatment. SBP scores were measured using the tail-cuff plethysmograph method (IITC/Life Science Instrument Model 229 and Model 179 amplifier: Woodland Hills, CA, USA). The average SBP value of three measurements was calculated.

### 2.4. Measurements of Fasting Blood Glucose, Serum Insulin Level, and the Oral Glucose Tolerance Test

All rats were allowed to receive the drinking water during the 12 h fast. Blood samples were collected from lateral tail veins to determine basal glycemic (at time 0 min (T0)) and serum insulin levels. Then, rats were fed with glucose solutions using a gavage tube at a dose of 2 g/kg BW. Blood glucose concentrations at 30, 60, 120, and 180 min after gavage were assessed using a glucometer (Roche Diagnostics GmbH, Mannheim, Germany). Insulin levels in serum were assessed after 12 h of fasting overnight. Serum samples were obtained upon spontaneous coagulation and centrifugation (3000 g, 4 °C, 30 min). Serum insulin concentrations were analyzed by enzyme-linked immunosorbent assay (ELISA) kits (Millipore Corporation, Billerica, MA, USA). Insulin resistance was determined from the relative value of homeostasis model (HOMA-IR) [43]. The HOMA-IR score was calculated using the formula as follows:

(1) $$\mathrm{HOMA}-\mathrm{IR}=\frac{\left(\mathrm{fasting}\;\mathrm{glucose}\;\left(\mathrm{mmol}/L\right)\right)\times \left(\mathrm{fasting}\;\mathrm{insulin}\;\left(\mu \mathrm{IU}/\mathrm{mL}\right)\right)}{22.5}$$

### 2.5. Echocardiography

At the 16th week, all rats were anesthetized with 3% isoflurane. Echocardiography was performed to measure cardiac function using a commercially available echocardiography system (Model LOGIQ S7), equipped with a 10 MHz linear transducer (GE Healthcare, WI, USA). Each rat was shaved around its chest, and a warmed resonance gel was applied to the hairless chest. The ultrasound transducer was placed slightly left of the chest and then optimized for the LV and aorta. Two-dimensional-guided M-mode images were recorded in accordance with the American Society of Echocardiography guidelines. Three consecutive beats were measured 5 min after anesthesia, and the average of these measurements was taken for analysis. M-mode tracings to record the interventricular septal end diastole and end systole (IVSd and IVSs), left ventricular internal diameter end diastole and end systole (LVIDd and LVIDs), left ventricular posterior wall end diastole and end systole (LVPWd and LVPWs), end-diastolic and systole volumes (EDV and ESV), stroke volume (SV), and ejection fraction (%EF) from three consecutive cardiac cycle were performed. The LV shortening fraction (%SF) was calculated using the equation %SF = [(LVIDd-LVIDs)/LVIDd] × 100.

### 2.6. Direct Blood Pressure Measurements

After echocardiography, the left femoral artery was cannulated and connected to a pressure transducer. Hemodynamic parameters such as SBP, diastolic blood pressure (DBP), mean arterial pressure (MAP), and heart rate (HR) were monitored and recorded by the Acknowledge Data Acquisition and Analysis Software (BIOPAC Systems Inc., Goleta, CA, USA).

### 2.7. Assessment of Biochemical Profiles

Following indirect blood pressure measurements, rats were euthanized by an overdose of anesthesia; then, blood samples were collected from the abdominal aorta, and the plasma was collected immediately by centrifugation at a speed of 3000 g at 4 °C for 30 min. The total cholesterol (TC), triglyceride (TG), and high-density lipoprotein cholesterol (HDL-C) levels in the plasma were determined spectrophotometrically using specific commercial kits (Human Gesellschaft für Biochemica and Diagnostica mbH, Wiesbaden, Germany). Additionally, liver tissue was homogenized in lysis buffer to measure TC and TG using specific commercial kits as plasma. Levels of aspartate transaminase (AST) and alanine transaminase (ALT) were determined by the Clinical Chemistry Laboratory Unit of the Faculty of Associated Medical Sciences, Khon Kaen University, Thailand.

### 2.8. Tissue Harvesting

After collecting blood samples, heart, liver, and visceral fat (including epididymal and retroperitoneal fats) were immediately dissected. All tissues were weighed to compare regional tissue weight (mg)/body weight (g). A portion of liver, heart, and visceral fat was frozen at −20 °C for biochemical analysis and fixed in 4% formaldehyde for histomorphology analysis.

### 2.9. Hematoxylin and Eosin Staining of Cardiac and Fat Tissue

Myocardial tissue and epididymal fat pads were fixed in 4% paraformaldehyde for 24 h, routinely processed, and embedded in paraffin. Briefly, all tissue paraffin blocks were cut to a 5 µm thickness using a microtome. The paraffin sections (5 µm) were dewaxed and rehydrated using gradient alcohol and water. The sections were then washed with tap water and distilled water and then stained with hematoxylin and eosin (H&E) (Bio-Optica Milano SpA., Milano, Italy). For microscopic assessment, the images of heart sections were captured by a stereoscope (Nikon SMZ745T with NIS-elements D 3.2) with a 1× objective lens to observe the LV wall thickness, the cross-sectional area (CSA), the LV luminal area, and the wall-to-lumen ratio. These parameters were counted using Image J software (National Institutes of Health, Bethesda, MD, USA).

The measurement of the myocardium cell size and the area of cardiomyocytes was performed for 300 myocytes per group with a 40× objective lens using a Digital sight DS-2MV light microscope (Nikon, Tokyo, Japan). Mean values were obtained for 300 cells/group.

Epididymal fat sections were evaluated using a Digital sight DS-2MV light microscope (Nikon, Tokyo, Japan) with a 40× objective lens. Adipocytes were quantitated according to the cell size area (300 cells/group) using NIS-Elements software.

### 2.10. Immunohistochemical Staining of Myocardial Sections

An immunohistochemical technique was used to evaluate TNF-α and IL-6 expression in the LV. The myocardial sections were deparaffinized in xylene and rehydrated through an ethanol series. Antigen retrieval was performed by tris-ethylenediaminetetraacetic acid (EDTA) buffer using the high-temperature heating method to recover the antigenicity of tissue sections. The myocardial sections were incubated with hydrogen peroxide (H_2_O_2_) to block endogenous peroxidase activity and then incubated with 5% bovine serum albumin in PBS to block nonspecific protein. Thereafter, the sections were incubated with primary antibody, mouse anti-TNF-α IgG (dilution 1:500), or mouse anti-IL-6 IgG (Catalog Number sc57315, dilution 1:500) in a moistening chamber for 4 h at room temperature (Santa Cruz Biotechnology, Inc., Santa Cruz, CA, USA). Goat anti-mouse IgG (HRP) at a dilution of 1:1000 (catalog number sc8436) (Abcam Plc, Cam-bridge, U.K.) was used as a secondary antibody. The brown color of 3,3′-diaminobenzidine (DAB) was visualized as a positive control, and the tissues were counterstained with hematoxylin. TNF-α and IL-6 expressions were counted using Image-Pro plus 6 software (Media Cybernetics, Inc., Rockville, MD, USA). In brief, the stained sections were captured under a Digital Sight DS-2MV light microscope (Nikon, Tokyo, Japan) with a 40× objective lens. Twelve images were randomly selected as representative images for the quantification of each sample using automatically counting thresholded pixels mode in Image-Pro plus 6 software. All evaluations were performed by one observer under intraobserver agreement. The levels of TNF-α and IL-6 were expressed as the percentage of the relative stained areas.

### 2.11. Assays of Cytokine Levels

The plasma adiponectin level was assessed using adiponectin enzyme-linked immunosorbent assay (ELISA) kits (Millipore Corporation, Billerica, MA, USA). The serum levels of TNF-α and IL-6 were measured with ELISA kits according to the manufacturer’s instructions (Sigma-Aldrich, Saint Louis, MO, USA).

### 2.12. Oxidative Stress Marker Assessment

The aorta was rapidly excised to determine superoxide generation using lucigenin-enhanced chemiluminescence as described previously [44]. The aorta was quickly dissected. The adherent fat and connective tissue were cleaned on ice. The vessel segments (3–5 mm) were placed in Krebs-KCl buffer and allowed to equilibrate at 37 °C for 30 min. Lucigenin was added to the sample tube and placed in a luminometer (Turner Biosystems, Sunnyvale, CA, USA). The photon counts were integrated every 30 s for 5 min. The vessels were dried at room temperature for 24 h to determine the dry weight. Superoxide production in the aorta was expressed as relative light unit counts per minute per milligram of dry tissue weight. Malondialdehyde (MDA) is an end-product of lipid peroxidation and can be used as a biomarker of oxidative damage. MDA was estimated in plasma and heart tissue by using a colorimetric assay or thiobarbituric acid reactive substance (TBARS) assay as described in a previous report [45]. The MDA level was assessed by quantifying thiobarbituric acid (TBA) reactivity as MDA in a spectrophotometer. The resulting chromogen absorbance was determined at a wavelength of 532 nm against a blank reference. The concentration of MDA was read from standard calibration curve plotted using 1, 1, 3, 3 tetra-ethoxy propane (TEP) as a μM/L unit.

### 2.13. Antioxidant Endogenous Enzyme Activity Assessment

The CAT activities in plasma and heart tissue were determined using a colorimetric method. CAT is a ubiquitous enzyme that destroys the hydrogen peroxide (H_2_O_2_) formed during oxidative stress. The level of CAT activity depends on the change of 405 nm absorbance at high levels of hydrogen peroxide solution. In brief, samples were incubated with substrate (65 µmol/mL of H_2_O_2_ in 60 mmol/L sodium potassium phosphate buffer pH 7.4) in a 96-well plate at 37 °C for 1 min. Next, we added 32.4 mmol/L ammonium molybdate to stop the reaction. The yellowish molybdate and H_2_O_2_ complex absorbance was determined at a wavelength of 405 nm to calculate the CAT activity level.

SOD activities in heart tissue were measured via colorimetric analysis using a spectrophotometer with the corresponding detection kits (Sigma-Aldrich, Merck KGaA, Darmstadt, Germany) according to the manufacturer’s protocols.

### 2.14. Western Blotting Analysis

LV tissue was homogenized in ice-cold lysis buffer. Processed samples, containing 50 μg protein, were heat denatured in Laemmli buffer and separated on 10% sodium dodecyl sulfate polyacrylamide gel (SDS-PAGE). Separated proteins were electro-transferred onto a polyvinylidene difluoride (PVDF) membrane (MilliporeSigma, Merck KGaA, Darmstadt, Germany) at 90 V for 90 min. After the completion of the transfer, the PVDF membranes were blocked with 5% BSA in tris-buffered saline with 0.1% Tween-20 (TBS-T) for 2 h at room temperature. After that, membranes were incubated overnight at 4 °C with specific primary antibodies against AdipoR1 (Catalog Number ab126611, dilution 1:1000), COX-2 (Catalog Number ab52237, dilution 1:500) (Abcam Plc, Cambridge, U.K.), and p-NF-κB (Catalog Number 3033S, dilution 1:1000) (Cell Signaling Technology, Inc., Danvers, USA). This was followed by incubation with an appropriate secondary antibody for 2 h at room temperature. β-actin was used as a loading control (Santa Cruz Biotechnology, Inc., Santa Cruz, CA, USA). Bands were detected using ECL^TM^ Prime Western blotting reagents (Amersham Biosciences Corp., Piscataway, NJ, USA). The intensities of the bands were quantified using an ImageQuant™ 600 imager (GE Healthcare Life Science, Piscataway, NJ, USA) and were normalized to that of β-actin.

### 2.15. Statistical Analysis

All results are shown as the mean ± standard error of the mean (S.E.M.). Data were analyzed by a one-way analysis of variance (ANOVA) followed by Tukey’s post hoc test for multiple comparisons analysis. The PRISM software Version 8.3 (GraphPad Software Inc., San Diego, CA, USA) was used to analyze the statistics. *p*-value <0.05 shows a significant difference.

## 3. Results

### 3.1. Effects of Galangin on Body and Organ Weight in Metabolic Syndrome Rats

After 16 weeks of the experiment, the final rat body weight did not differ between the control group and MS group (*p* < 0.05). Additionally, the weights of the whole heart, ventricles, retroperitoneal fat pads, epididymal fat pads, and liver were significantly higher in MS rats than those of the control group (*p* < 0.05). Moreover, MS rats showed significant increases in the ratio of retroperitoneal fat pads/body weight and epididymal fat pads/body weight compared to the control group (*p* < 0.05) (Table 1.). Treatment with galangin did not reduce the body and organ weight in MS rats compared to the untreated group. Liver weight loss was shown in the galangin-treated group (50 mg/kg) and metformin-treated group (*p* < 0.05). In addition, metformin treatment alleviated the weight of the whole heart, ventricular weight, retroperitoneal fat pads, and retroperitoneal fat pads/body weight (*p* < 0.05), as shown in Table 1.

### 3.2. Effects of Galangin on Metabolic Parameters

**Figure 1:**
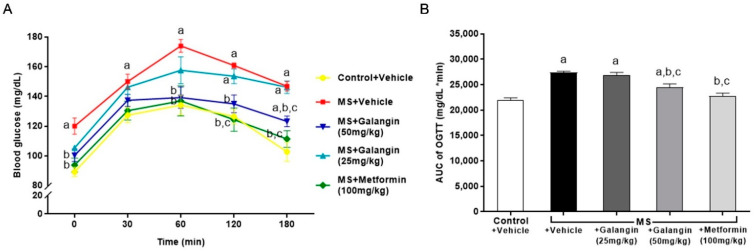
Effects of galangin and metformin treatments on blood glucose concentrations: (**A**) oral glucose tolerance test (OGTT) and (**B**) and area under the curve (AUC) of OGTT. Data are presented as the mean ± S.E.M. (*n* = 8). ^a^ *p* < 0.05 vs. the control group, ^b^ *p* < 0.05 vs. the MS group, and ^c^ *p* < 0.05 vs. MS + the galangin (25 mg/kg) group.

The baseline blood glucose level (T = 0 min) was high in HF diet-fed rats compared to the control rats. The blood glucose levels of all the rats peaked at 60 min, and thereafter, the blood glucose concentrations decreased. The blood glucose levels in the MS rats did not recover to the baseline levels at 120 min, while the levels of blood glucose in the MS rats treated with galangin (50 mg/kg) or metformin did not differ from those in the control group by 120 min (Figure 1A) (*p* < 0.05). The areas under the curve (AUCs) of OGTT were larger in the MS rats, and this was attenuated in the MS rats treated with galangin or metformin (Figure 1A).

It was found that fasting blood glucose, fasting insulin, and the HOMA-IR index were higher in the MS group than the control group (*p* < 0.05). Galangin (50 mg/kg) and metformin corrected the insulin resistance by reducing the levels of fasting glucose, fasting insulin, and the HOMA-IR index in MS rats (*p* < 0.05) (Table 2.). Rats fed with an HF diet had an apparent elevation of total cholesterol and triglyceride in plasma and liver tissue, as compared to the control group. However, a reduction of plasma HDL-C was found in the MS group (*p* < 0.05). The liver enzymes, both plasma aspartate transaminase (AST) and alanine transaminase (ALT), were significantly higher in the MS group than the control group (*p* < 0.05). Galangin was effective at improving the levels of total cholesterol, triglyceride, HDL-C, AST, and ALT in rats with HF diet-induced MS in a dose-dependent manner. Metformin also ameliorated the disturbance of all metabolic parameters in MS rats (Table 2).

### 3.3. Effects of Galangin on Epididymal Fat Pads’ Morphology

**Figure 2:**
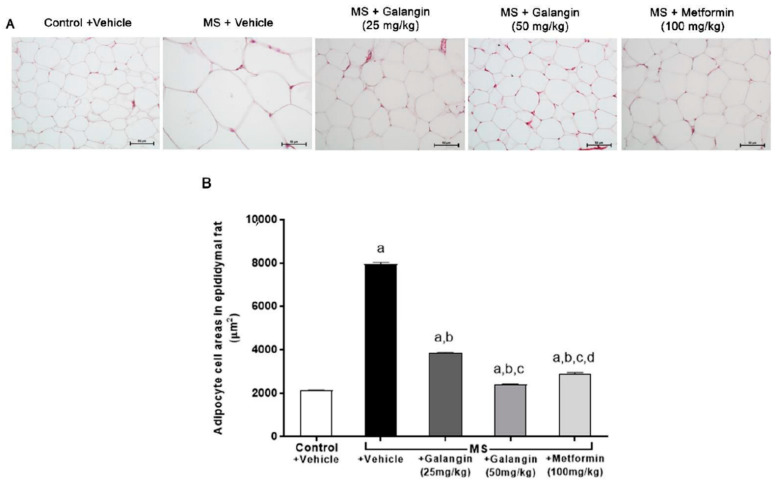
Morphology of epididymal fat pads. (**A**) Representative photographs of epididymal fat sections stained with hematoxylin and eosin (H&E) (magnification ×200, scale bar = 50 µm). (**B**) Effects of galangin and metformin treatments on adipocyte cell areas. Data are presented as the mean ± S.E.M. (*n* = 8). ^a^ *p* < 0.05 vs. the control group, ^b^ *p* < 0.05 vs. the MS group, ^c^ *p* < 0.05 vs. the MS + galangin (25 mg/kg) group, and ^d^ *p* < 0.05 vs. the MS + galangin (50 mg/kg) group.

The histological findings, as shown in Figure 2, revealed the hypertrophy of adipocytes from epididymal fat pads in the MS group compared with the control group. Galangin (25 or 50 mg/kg) and metformin treatments reduced the hypertrophy of adipocytes compared to the untreated MS group (*p* < 0.05), as shown in Figure 2A,B.

### 3.4. Effects of Galangin on Blood Pressure and Heart Rate

**Figure 3:**
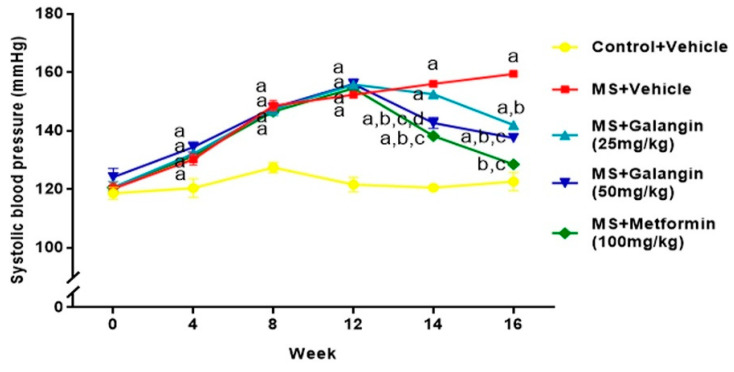
Effects of galangin and metformin treatments on systolic blood pressure. Data are presented as the mean ± S.E.M. (*n* = 8). ^a^ *p* < 0.05 vs. the control group, ^b^ *p* < 0.05 vs. the MS group, ^c^ *p* < 0.05 vs. the MS + galangin (25 mg/kg) group, and ^d^ *p* < 0.05 vs. the MS + galangin (50 mg/kg) group.

Rats receiving an HF diet for four weeks had high blood pressure compared to the control rats (*p* < 0.05). After 16 weeks of the experiment, untreated MS rats showed hypertension compared to the control group (SBP = 156.10 ± 0.75 vs. 120.50 ± 1.10 mmHg,) (*p* < 0.05). Galangin (25 or 50 mg/kg) administrations for four weeks significantly reduced the elevation of systolic blood pressure (SBP = 142.00 ± 0.60 or 137.57 ± 1.25 mmHg) in MS rats (*p* < 0.05). Metformin restored the blood pressure in MS rats to close to the level in normal rats (SBP = 128.50 ± 0.53 mmHg, *p* < 0.05) (Figure 3). Furthermore, the SBP values in all rats measured by an indirect method were consistent with the result of hemodynamic parameters evaluated by a direct method, as shown in Table 3. The heart rate of the MS group was significantly higher than that of the control group, and this was suppressed in MS rats treated with galangin (25 or 50 mg/kg) or metformin (*p* < 0.05) (Table 3).

### 3.5. Effects of Galangin on Cardiac Function Parameters

Echocardiography revealed decreased LVIDd and increased LVPWd in the MS group compared to the normal rats (*p* < 0.05). The values of cardiac performance parameters including EDV, SV, EF, and FS were significantly decreased in MS rats compared to those of the control group (*p* < 0.05). These impairments of cardiac function were alleviated in galangin and metformin administrations (*p* < 0.05) in MS rats compared to control rats, as shown in Table 4.

### 3.6. Effects of Galangin on Cardiac Morphology

**Figure 4:**
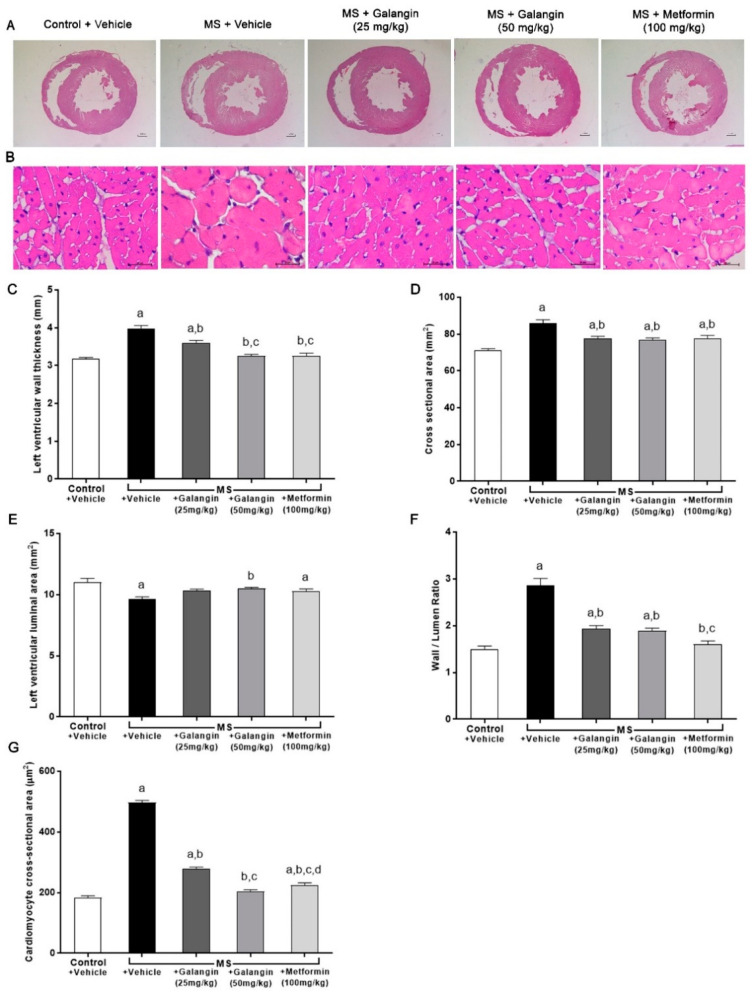
Morphology of heart. (**A**) Representative photographs of heart sections stained with H&E (magnification ×10) (scale bar = 5 mm). (**B**) Representative cross-sections of cardiomyocytes stained with H&E (magnification ×400) (scale bar = 20 µm). (**C**) Effects of galangin and metformin treatments on left ventricular wall thickness, (**D**) cross-sectional areas, (**E**) left ventricular luminal areas, (**F**) wall/lumen ratio, and (**G**) cardiomyocyte area. Results are shown as the mean ± S.E.M. (*n* = 8). ^a^ *p* < 0.05 vs. the control group, ^b^ *p* < 0.05 vs. the MS group, ^c^ *p* < 0.05 vs. the MS + galangin (25 mg/kg) group, and ^d^ *p* < 0.05 vs. the MS + galangin (50 mg/kg) group.

Morphological changes in LV were observed in rats with MS induced by an HF diet. Significant increases in the LV wall thickness, CSA, and wall/lumen ratio and a reduction of the LV luminal areas were observed in the MS group (*p* < 0.05). This cardiac hypertrophy was consistent with the appearance of cardiomyocytes since there was a significant increase in the cell size of cardiomyocytes in the MS group. Galangin (25 or 50 mg/kg) and metformin treatments significantly reduced cardiac hypertrophy and cell size in MS rats compared to the untreated group (*p* < 0.05) (Figure 4C–G).

### 3.7. Effects of Galangin on Myocardial Inflammation

**Figure 5:**
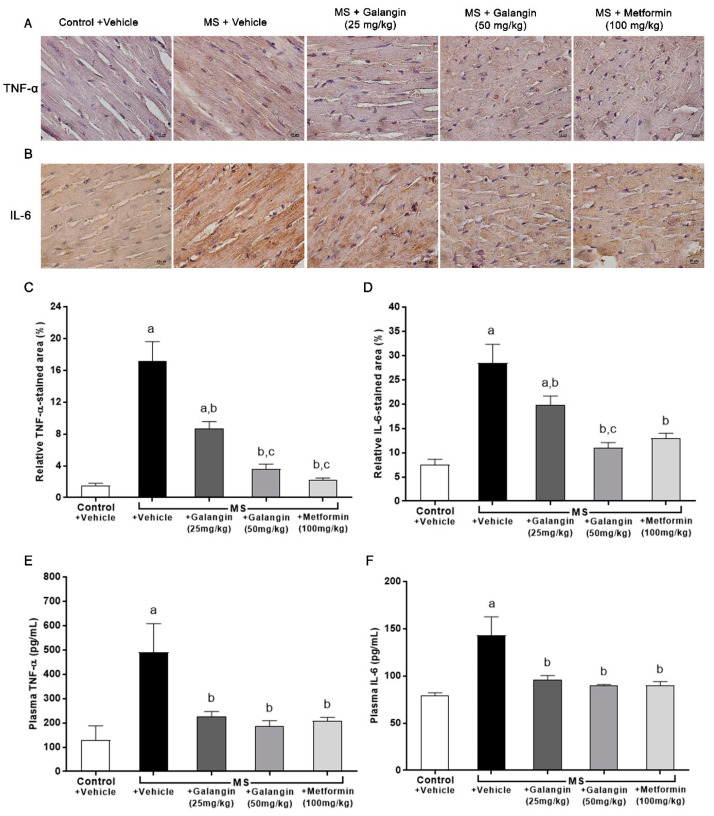
Effects of galangin and metformin treatments on tumor necrosis factor-α (TNF-α) and interleukin-6 (IL-6) immunohistochemical staining for myocardial and TNF-α and IL-6 levels. (**A**) TNF-α, (**B**) IL-6 (Brown Chromogen) immunohistochemical staining in myocardia (magnification × 400) (scale bar = 10 µm), (**C**) relative TNF-α-stained area (%), (**D**) relative IL-6-stained area (%), (**E**) plasma TNF-α, and (**F**) plasma IL-6. Results are shown as the mean ± S.E.M. (*n* = 8). ^a^ *p* < 0.05 vs. the control group, ^b^ *p* < 0.05 vs. the MS group, and ^c^ *p* < 0.05 vs. the MS + galangin (25 mg/kg) group.

The expressions of inflammatory mediator proteins TNF-α and IL-6 were enhanced, as shown in Figure 5A,B. Notably, the immunohistochemical staining revealed that galangin (25 or 50 mg/kg) and metformin treatment suppressed the expression of TNF-α and IL-6 (*p* < 0.05) (Figure 5C,D). The plasma concentrations of TNF-α and IL-6 were significantly raised in the MS group compared with the control group (*p* < 0.05). In turn, galangin and metformin treatment alleviated the high concentrations of plasma inflammatory cytokines in MS rats as compared to untreated rats (*p* < 0.05) (Figure 5E,F).

### 3.8. Effects of Galangin on Plasma Adiponectin Levels

**Figure 6:**
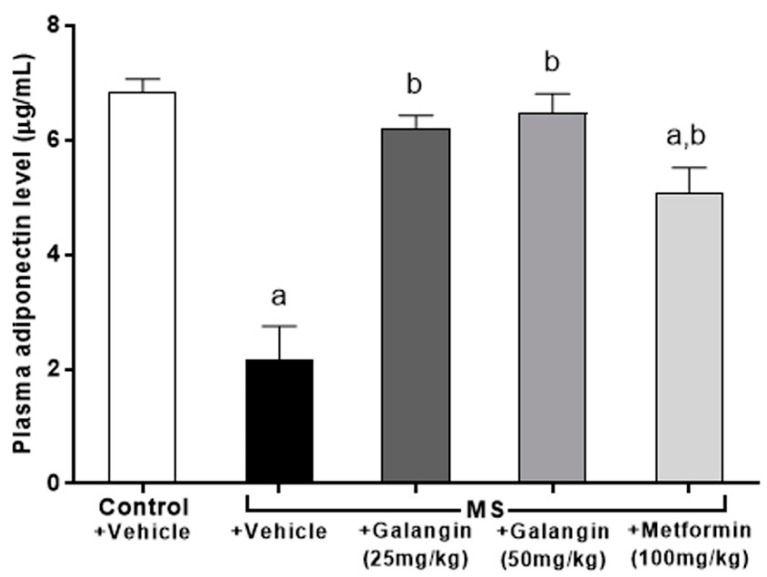
Effects of galangin and metformin treatments on plasma adiponectin levels. Results are shown as the mean ± S.E.M. (*n* = 8). ^a^ *p* < 0.05 vs. the control group and ^b^ *p* < 0.05 vs. the MS group.

Adiponectin concentrations were low in the MS group compared to the control group (*p* < 0.05). Rats that were treated with galangin (25 or 50 mg/kg) or metformin, however, showed an inverse change in adiponectin concentration compared to the MS rats (Figure 6).

### 3.9. Effects of Galangin on Oxidative Stress Markers and Endogenous Antioxidant Enzymes

**Figure 7:**
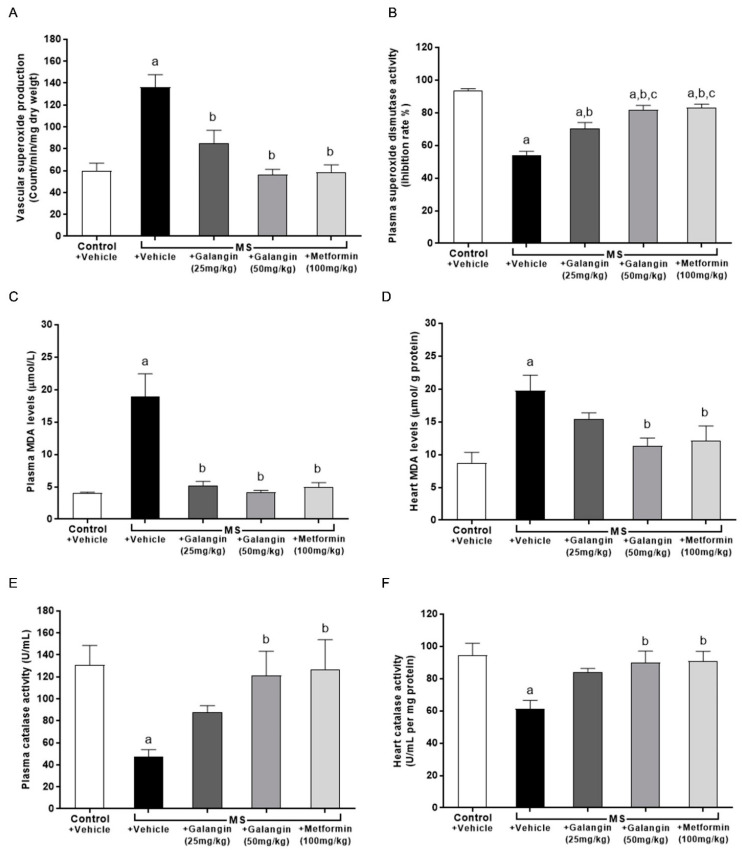
Effects of galangin and metformin treatments on oxidative stress markers and endogenous antioxidant enzymes. (**A**) Aortic superoxide production, (**B**) plasma malondialdehyde (MDA) level, (**C**) heart MDA level (**D**) plasma superoxide dismutase (SOD) activity, (**E**) plasma catalase (CAT) activity, and (**F**) heart CAT activity. Results are shown as the mean ± S.E.M. (*n* = 8). ^a^ *p* < 0.05 vs. the control group, ^b^ *p* < 0.05 vs. the MS group, and ^c^ *p* < 0.05 vs. the MS + galangin (25 mL/kg) group.

The results showed that superoxide production in the aorta was significantly elevated in the MS group compared with the control group (*p* < 0.05) (Figure 7A). The levels of MDA in plasma and cardiac tissue were raised in the MS group, and these were significantly attenuated by galangin (25 or 50 mg/kg) and metformin administrations compared with the untreated MS group (*p* < 0.05) (Figure 7B,C). CAT activities were decreased in the plasma and heart tissue in the MS group compared with the control group (*p* < 0.05) (Figure 7E,F). SOD activities were lower in MS rats than control rats. Galangin (25 or 50 mg/kg) and metformin treatment in MS rats significantly enhanced the CAT and SOD activities in MS rats (*p* < 0.05) (Figure 7D–F).

### 3.10. Effects of Galangin on AdipoR1, COX-2, and p-NF-κB Expression in Cardiac Tissue

**Figure 8:**
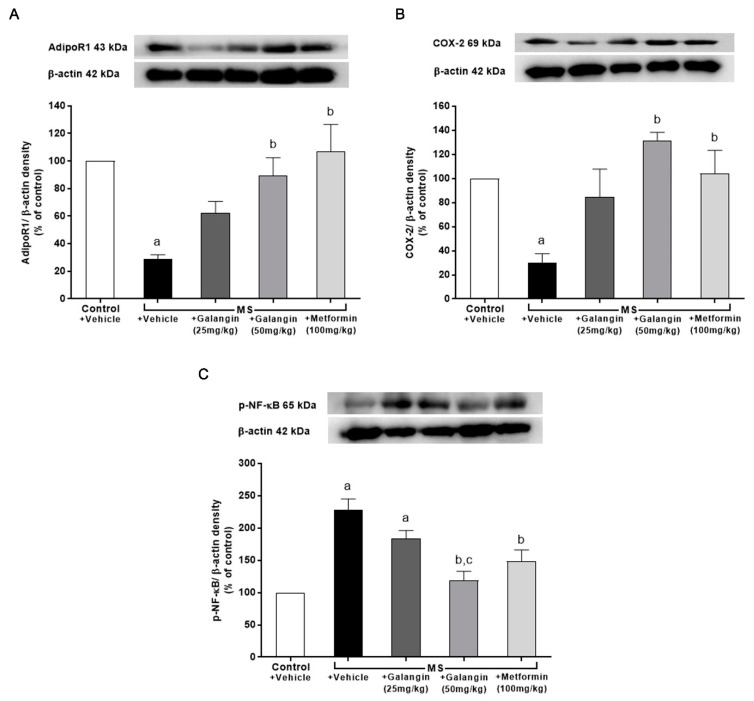
Effects of galangin and metformin treatments on protein expression in heart: (**A**) AdipoR1, (**B**) COX-2, and (**C**) p-NF-κB. Data are presented as the mean ± S.E.M. (*n* = 3–4). ^a^ *p* < 0.05 vs. the control group and ^b^ *p* < 0.05 vs. the MS group. ^c^ *p* < 0.05 vs. MS + Galangin (25 mL/kg) group. AdipoR1: adiponectin receptor 1; COX-2: cyclooxygenase-2; p-NF-κB: phospho-nuclear factor kappa B.

The downregulation of AdipoR1 and COX-2 expressions in cardiac tissue in the MS rats was observed; however, the expression of *p*-NF-κB was significantly upregulated in the MS group (*p* < 0.05). Meanwhile, the MS rats treated with galangin and metformin at a dose of 50 mg/kg exhibited significantly improved expression of AdipoR1, COX-2, and p-NF-κB (*p* < 0.05) (Figure 8).

## 4. Discussion

The results of this study showed that galangin alleviated signs of MS, dyslipidemia, hyperglycemia, insulin resistance, visceral fat accumulation, and hypertension in rats with an HF diet plus 15% fructose-induced MS. LV remodeling and dysfunction were shown in MS rats and regressed in the galangin-treated group. High concentrations of the proinflammatory cytokines TNF-α and IL-6 in plasma and myocardium and low concentrations of plasma adiponectin were found in rats receiving an HF diet, and these were alleviated in the galangin-treated group. Galangin showed antioxidant activity by decreasing plasma and cardiac MDA and aortic superoxide production and increasing endogenous antioxidant enzyme activities in MS rats. Galangin also restored the alterations of AdipoR1, COX-2 and NF-κB protein expression in the cardiac tissue of MS rats. Metformin ameliorated signs of MS, cardiac abnormalities, inflammation, and oxidative stress in MS rats.

Many studies have presented an animal model of MS induced by an HF diet that was characterized by augmented body weight gain, hyperglycemia, hyperinsulinemia, hypertension, and dyslipidemia [46]. In this study, rats fed an HF diet and fructose developed MS together with an enlargement of adipocytes and high levels of liver enzymes. An impairment of liver function has been reported in MS rats induced by an HF diet that resulted from fat accumulation, insulin resistance, oxidative stress, and inflammation [46]. Fructose is an important factor to facilitate the development of MS since hepatic fructose uptake is independent of energy status, leading to increased lipogenesis [47]. High blood pressure is the consequence of insulin resistance induced by a high-fat diet and fructose, as has been noted previously [48]. Furthermore, the impairment of cardiac function including decreased EDV, SV, and FS was observed in rats fed an HF diet. These results were supported by previous studies [9,27]. In the present study, it could be described that cardiac dysfunction in MS rats was the consequence of cardiac remodeling, as evidenced by increases in LV wall thickness, cross-sectional areas, wall/lumen ratio, and cardiomyocyte area, as well as a decrease in LV luminal areas. Increased LVPWd and decreased LVIDd were consistent with the results of LV hypertrophy. Generally, there are at least two factors—mechanical or pressure overload and humoral agents—that stimulate cardiac remodeling [49,50]. Furthermore, inflammation has been established as the main process of the myocardial remodeling of the adaptive responses of heart to stress [51]. Numerous reports have illustrated that cardiac changes were associated with obesity, hyperinsulinemia, impaired glycemic control, dyslipidemia, inflammation, and oxidative stress [52]. The results of this study showed local and systemic inflammation, as indicated by increases in the cardiac and plasma pro-inflammatory cytokines IL-6 and TNF-α. In this study, oxidative stress occurred in rats fed an HF diet as a consequence of increasing aortic superoxide production and cardiac and plasma MDA levels, as well as decreasing endogenous antioxidant enzyme activities. These results were supported by the evidence that the long-term consumption of an HF diet promoted oxidative stress and inflammation, activating stimulated fibrotic remodeling in heart [53].

Adiponectin is an anti-inflammatory cytokine and exerts cardioprotective effects [16]. In animal models of ischemia–reperfusion (I/R)-induced cardiac injury, adiponectin decreased infarct size and alleviated cardiac dysfunction, which was associated with reducing inflammation, apoptosis, and oxidative stress [20,54]. This study found that the concentrations of serum adiponectin were low in rats fed an HF diet. This result was consistent with other previous studies showing that adiponectin has a crucial role in the regulation of metabolic parameters to increase insulin sensitivity [55,56,57]. In the cardiac tissue of rats fed an HF diet, the expression of AdipoR1 and COX-2 protein decreased while an upregulation of the expression of NF-κB was observed. The dualistic effect of COX-2 on obesity and insulin resistance, as well as the pathogenesis of cardiovascular events, however, has been noted [58]. The beneficial effect of COX-2 in an HF diet-induced MS in this study was supported by the evidence that COX-2 was required for de novo recruitment of brown adipose tissues in white adipose tissues to enhance thermogenesis and systemic energy expenditure and to prevent HF diet-induced obesity and insulin resistance in mice [59]. Hepatic expression of COX-2 can prevent HF diet-induced hepatic steatosis, dyslipidemia, insulin resistance, and obesity [60]. The aforementioned actions of COX-2 are primarily associated with its products, prostaglandins. Furthermore, the myocardial protection of COX-2 mediated by prostaglandin E2 and prostacyclin has also been demonstrated [21]. In contrast, accumulating evidence showed that COX-2-derived prostaglandins play a detrimental role in the development of obesity, cardiovascular disease, and insulin resistance [58]. The protective effect of adiponectin on myocardial I/R injury might be involved in its ability to stimulate COX-2 in cardiac cells [20]. Shibata and coworkers demonstrated that adiponectin also increased the expression of COX-2, which inhibits TNF-α production in myocytes. The protein expression of NF-κB in the present study was consistent with the expression of TNF-α and IL-6 in cardiac tissue, as well as their high levels in plasma. It is possible that the cardiac remodeling that occurred in rats fed an HF diet could be relevant to the protein expressions of AdipoR1, COX-2, and NF-κB.

Galangin alleviated cardiometabolic alterations in rats with an HF diet-induced MS, as evidenced by resolving hyperglycemia, impaired glucose tolerance, insulin resistance, dyslipidemia, visceral fat deposition, and hypertension. Galangin reduced the enlargement of adipocytes and levels of hepatic enzymes. It also improved cardiac function and regressed cardiac remodeling in MS rats. Our results were in accordance with previous studies showing that galangin decreased adipose tissue and liver weight in cafeteria-diet-fed female rats [61]. It has been reported to improve blood glucose, total cholesterol, triglyceride, and HDL-c in the plasma and liver of STZ-induced hyperglycemia and cafeteria-diet-fed female rats [41,61]. The effect of galangin on cardiometabolic disorders in the present study might be related to biomarkers reducing systemic oxidative stress in vessels and heart and increasing endogenous antioxidant activities. An anti-oxidative effect of galangin has been strongly proposed [40]. Additionally, the anti-inflammatory effects of galangin shown in this study, supported by reductions in heart and systemic concentrations of TNF-α and IL-6, might enhance its beneficial effects on heart. Serum adiponectin levels were increased by galangin treatment in rats fed an HF diet, and this might suppress inflammation in heart and the circulatory system. Galangin also increased the expression of AdipoR1 and COX-2 and suppressed the expression of NF-κB expressions in cardiac tissue. It is well established that NF-κB is associated with inflammatory responses [62]. In contrast, COX-2 has been reported to alleviate heart failure in late ischemic preconditioning [63,64]. These data could suggest that galangin alleviated cardiometabolic disorders associated with oxidative stress and inflammation via the restoration of AdipoR1, COX-2, and NF-κB protein expressions in heart. Metformin suppressed signs of MS and alleviated cardiac changes in rats fed an HF diet. It also reduced oxidative stress and inflammation relevant to the modulation of AdipoR1, COX-2, and NF-κB protein expressions in heart. These results confirmed that metformin is a hypoglycemic agent [31]. The cardioprotective effects of metformin have been reported in patients and experimental rats [65,66]. It also exhibited anti-inflammatory and antioxidant effects [33,34]. The level of adiponectin was increased by metformin in rats fed an HF diet, which was in agreement with a study showing that metformin treatment increased plasma adiponectin concentrations in obesity and type 2 diabetes [67,68].

## 5. Conclusions

In conclusion, these data suggest that galangin resolved cardiometabolic disorders and LV dysfunction and remodeling induced by an HF diet plus fructose in rats, as it was involved in reduced inflammation and oxidative stress.

## Figures and Tables

**Table 1:** Effects of galangin and metformin on body and organ weight in metabolic syndrome rats.

	Control + Vehicle	MS + Vehicle	MS + Galangin (25 mg/kg)	MS + Galangin (50 mg/kg)	MS + Metformin (100 mg/kg)
BW (g)	714.17 ± 22.69	837.14 ± 35.59 ^a^	793.29 ± 17.75	789.50 ± 16.37	739.13 ± 28.12
HW (g)	1.47 ± 0.05	1.76 ± 0.06 ^a^	1.72 ± 0.04 ^a^	1.59 ± 0.04	1.54 ± 0.06 ^b^
HW/BW (mg/g)	2.06 ± 0.05	2.11 ± 0.07	2.17 ± 0.02	2.01 ± 0.06	2.09 ± 0.05
VW (g)	1.30 ± 0.04	1.52 ± 0.05 ^a^	1.44 ± 0.02	1.41 ± 0.03	1.32 ± 0.05 ^b^
VW/BW (mg/g)	1.82 ± 0.03	1.84 ± 0.04	1.82 ± 0.02	1.79 ± 0.03	1.80 ± 0.04
RP pads weight (g)	18.96 ± 2.66	59.91 ± 5.23 ^a^	55.62 ± 3.13 ^a^	52.25 ± 1.24 ^a^	40.53 ± 6.13 ^a,b^
RP pads weight/BW (mg/g)	26.43 ± 3.35	70.86 ± 3.57 ^a^	69.85 ± 2.84 ^a^	67.00 ± 1.00 ^a^	53.54 ± 6.63 ^a,b^
EP pads weight (g)	14.81 ± 0.72	29.09 ± 2.85 ^a^	26.13 ± 0.76 ^a^	25.52 ± 1.94 ^a^	23.85 ± 2.68 ^a^
EP pads weight/BW (mg/g)	20.76 ± 0.88	34.39 ± 2.09 ^a^	33.15 ± 1.15 ^a^	32.62 ± 1.89 ^a^	31.81 ± 3.09 ^a^
LW(g)	19.01 ± 1.17	24.34 ± 1.20 ^a^	21.74 ± 0.92	19.72 ± 0.70 ^b^	19.79 ± 0.75 ^b^
LW/BW (mg/g)	26.81 ± 1.92	29.00 ± 1.03	27.41 ± 0.95	25.34 ± 0.51	26.85 ± 0.86

Results are shown as the mean ± S.E.M. (*n* = 8). ^a^ *p* < 0.05 vs. the control group, and ^b^ *p* < 0.05 vs. the MS group. MS: metabolic syndrome; BW: body weight; HW: heart weight; VW: ventricular weight; RP: retroperitoneal fat; EP: epididymal fat; and LW: liver weight.

**Table 2:** Effects of galangin and metformin treatments on metabolic parameters in rats with high-fat diet-induced metabolic syndrome.

	Control + Vehicle	MS + Vehicle	MS + Galangin (25 mg/kg)	MS + Galangin (50 mg/kg)	MS + Metformin (100 mg/kg)
Fasting blood glucose (mg/dL)	89.20 ± 3.18	120.00 ± 5.42 ^a^	105.40 ± 1.33	100.40 ± 4.17 ^b^	93.80 ± 4.65 ^b^
Fasting serum insulin (ng/mL)	1.99 ± 0.26	4.02 ± 0.71 ^a^	2.76 ± 0.25	2.06 ± 0.52 ^b^	2.13 ± 0.25 ^b^
HOMA-IR index	10.87 ± 1.38	30.26 ± 6.95 ^a^	17.73 ± 1.38	12.49 ± 3.12 ^b^	12.33 ± 1.89 ^b^
Plasma total cholesterol (mmol/L)	0.92 ± 0.03	2.57 ± 0.30 ^a^	1.35 ± 0.08 ^b^	1.00 ± 0.10 ^b^	1.17 ± 0.07 ^b^
Plasma triglyceride (mmol/L)	0.41 ± 0.07	2.49 ± 0.43 ^a^	0.71 ± 0.19 ^b^	0.54 ± 0.09 ^b^	0.45 ± 0.06 ^b^
Plasma HDL-C (mmol/L)	1.71 ± 0.13	0.33 ± 0.03 ^a^	0.79 ± 0.09 ^a,b^	1.30 ± 0.15 ^b,c^	0.99 ± 0.11 ^a,b^
Liver total cholesterol content (mg/g tissue)	11.17 ± 1.87	23.87 ± 2.71 ^a^	17.64 ± 1.46	11.53 ± 1.57 ^b^	11.58 ± 2.96 ^b^
Liver triglyceride content (mg/g tissue)	13.79 ± 1.10	33.13 ± 1.58 ^a^	19.42 ± 1.32 ^b^	16.29 ± 2.26 ^b^	15.42 ± 1.71 ^b^
AST (U/L)	49.00 ± 4.63	83.55 ± 4.20 ^a^	76.17 ± 4.56 ^a^	52.20 ± 1.43 ^b,c^	65.50 ± 3.59 ^a,b^
ALT (U/L)	19.13 ± 2.88	39.60 ± 2.58 ^a^	29.17 ± 3.20 ^b^	22.86 ± 0.86 ^b^	24.75 ± 1.66 ^b^

Results are presented as the mean ± S.E.M. (*n* = 8). ^a^ *p* < 0.05 vs. the control group, ^b^ *p* < 0.05 vs. the MS group, and ^c^ *p* < 0.05 vs. the MS + galangin (25 mg/kg) group. MS: metabolic syndrome; HDL-C: high-density lipoprotein cholesterol; AST: aspartate transaminase; ALT: alanine transaminase; HOMA-IR: relative value of homeostasis model.

**Table 3:** Effects of galangin on blood pressure obtained from a direct method of blood pressure measurement in rats with high-fat diet-induced metabolic syndrome.

	Control + Vehicle	MS + Vehicle	MS + Galangin (25 mg/kg)	MS + Galangin (50 mg/kg)	MS + Metformin (100 mg/kg)
Systolic blood pressure (mmHg)	118.49 ± 2.50	156.78 ± 1.96 ^a^	146.71 ± 2.24 ^a^	131.41 ± 2.75 ^a,b,c^	120.86 ± 3.04 ^b,c,d^
Diastolic blood pressure (mmHg)	80.28 ± 3.06	107.32 ± 1.11 ^a^	97.46 ± 3.12 ^a^	85.72 ± 2.00 ^b,c^	75.24 ± 3.43 ^b,c^
Mean arterial pressure (mmHg)	93.58 ± 2.82	123.90 ± 1.19 ^a^	113.73 ± 2.55 ^a^	100.95 ± 2.05 ^a,b,c^	90.48 ± 2.78 ^b,c^
Pulse pressure	39.89 ± 1.67	49.73 ± 2.11	48.81 ± 2.99	45.68 ± 2.10	45.72 ± 3.81
Heart rate (bmp)	331.86 ± 6.06	378.57 ± 9.84 ^a^	336.15 ± 11.29 ^b^	336.09 ± 9.99 ^b^	314.95 ± 4.68 ^b^

Results are presented as the mean ± S.E.M. (*n* = 8). ^a^ *p* < 0.05 vs. the control group, ^b^ *p* < 0.05 vs. the MS group, ^c^ *p* < 0.05 vs. the MS + galangin (25 mg/kg) group, and ^d^ *p* < 0.05 vs. the MS + galangin (50 mg/kg) group.

**Table 4:** Effects of galangin and metformin treatments on transthoracic echocardiographic parameters in rats with high-fat diet-induced metabolic syndrome.

	Control + Vehicle	MS + Vehicle	MS + Galangin (25 mg/kg)	MS + Galangin (50 mg/kg)	MS + Metformin (100 mg/kg)
IVSd (cm)	0.178 ± 0.005	0.200 ± 0.008	0.177 ± 0.008	0.174 ± 0.012	0.180 ± 0.008
IVSs (cm)	0.273 ± 0.010	0.272 ± 0.015	0.271 ± 0.10	0.283 ± 0.010	0.281 ± 0.012
LVIDd (cm)	0.759 ± 0.018	0.626 ± 0.030 ^a^	0.793 ± 0.018 ^b^	0.776 ± 0.026 ^b^	0.747 ± 0.008 ^b^
LVIDs (cm)	0.463 ± 0.023	0.434 ± 0.024	0.501 ± 0.020	0.463 ± 0.014	0.459 ± 0.008
LVPWd (cm)	0.197 ± 0.010	0.244 ± 0.013 ^a^	0.191 ± 0.011 ^b^	0.190 ± 0.010 ^b^	0.198 ± 0.006 ^b^
LVPWs (cm)	0.278 ± 0.009	0.307 ± 0.014	0.279 ± 0.008	0.277 ± 0.009	0.285 ± 0.009
EDV (mL)	0.969 ± 0.061	0.586 ± 0.077 ^a^	1.096 ± 0.070 ^b^	1.036 ± 0.092 ^b^	0.930 ± 0.029 ^b^
ESV (mL)	0.252 ± 0.031	0.212 ± 0.030	0.314 ± 0.037	0.249 ± 0.020	0.238 ± 0.013
EF (%)	74.480 ± 1.880	63.591 ± 3.185 ^a^	71.683 ± 1.417	75.839 ± 0.928 ^b^	74.093 ± 1.416 ^b^
SV (mL)	0.717 ± 0.033	0.374 ± 0.058 ^a^	0.780 ± 0.034 ^b^	0.790 ± 0.074 ^b^	0.689 ± 0.029 ^b^
FS (%)	38.840 ± 1.680	30.518 ± 2.137 ^a^	36.854 ± 0.972 ^b^	39.859 ± 0.851 ^b^	38.369 ± 1.204 ^b^

Results are presented as the mean ± S.E.M. (*n* = 8). ^a^ *p* < 0.05 vs. the control group and ^b^ *p* < 0.05 vs. the MS group. MS: metabolic syndrome; IVSd; interventricular septal at end diastole; IVSs: interventricular septal at end systole; LVIDd: left ventricular internal dimension at end-diastole; LVIDs: left ventricular internal dimension at end-systole; LVPWd: left ventricular posterior wall at end diastole; LVPWs: left ventricular posterior wall at end systole; EDV: end-diastolic volumes; ESV: end-systolic volumes; EF: ejection fraction; SV: stroke volume; FS: fractional shortening.

## Data Availability

No new data were created or analyzed in this study.
